# Frontline staff experiences of bridging dual diagnosis treatments – Determinants for implementing a cross-sectoral collaboration model

**DOI:** 10.1186/s13722-026-00681-3

**Published:** 2026-05-29

**Authors:** Ditte Maria Sivertsen, Signe Wegmann Düring, Katrine Schepelern Johansen, Jeanette Wassar Kirk

**Affiliations:** 1 Clinical Academic Group, Mental Health Services of the Capital Region, Copenhagen University Hospital, Copenhagen, Denmark; 2 Psychiatric Research Unit, Region Zealand, Slagelse, Denmark; 3 Department of Clinical Medicine, University of Copenhagen, Copenhagen, Denmark; 4 Department of Clinical Research, Copenhagen University Hospital, Hvidovre, Copenhagen, Denmark; 5 National Institute of Public Health, University of Southern Denmark, Copenhagen, Denmark

**Keywords:** Barriers, Cross-sector, Determinants, Dual diagnosis, Facilitators, Implementation, Substance use, Psychiatry, Qualitative.

## Abstract

**Background:**

It is estimated that 30–70% of patients in psychiatry have co-existing psychiatric disorders and substance use disorders, also called dual diagnosis. This condition often results in a complex clinical condition that affects the treatment trajectory and outcomes. Although the two conditions are intertwined, they are handled in two different sectors in Denmark, and therefore mostly treated separately. To overcome this divide, a coordination model was developed, with the aim of coordinating treatment between psychiatric outpatient FACT teams and municipal substance use treatment facilities for patients with dual diagnosis. The aim of this study was to assess the barriers and facilitators to a cross-sectoral coordination model as perceived by frontline staff during implementation.

**Methods:**

24 semi-structured interviews with frontline staff were performed in both sectors. The interview guide and analysis are based on the *Consolidated Framework for Implementation Research* (CFIR) in combination with *Qualitative Content Analysis*.

**Results:**

During implementation of the coordination model, frontline staff perceived barriers and facilitators in outpatient psychiatry and substance use treatment centers within all domains of CFIR, yet overall, participants were positive about the benefits and potentials of the model. During implementation they experienced an improvement in coordination practices, although patients were still rejected despite use of implementation strategies aimed at knowledge and skills, staff in both sectors did not feel competent to treat the target group and called for more training and concrete action plans. The flexibility required by this target group was considered hard to attain due to a lack of time and staff resources.

**Conclusion:**

According to frontline staff, a sustainable effort and stronger cross-sector collaboration calls for extensive management support, an ongoing focus on culture- and behavior changes, and enhancement of competences associated with addressing, detecting, and treating dual diagnosis.

**Supplementary Information:**

The online version contains supplementary material available at 10.1186/s13722-026-00681-3.

## Background

Dual diagnosis is a substantial health challenge that has consequences reaching far beyond psychiatry - into somatic health care and social arenas in everyday lives [1–3]. The term ‘dual diagnosis’ refers to *“the co-occurrence in the same individual of a psychoactive substance use disorder and another psychiatric disorder”* [4]. However, consensus regarding the term has not been reached in academia, where numerous alternative terms are being used, such as co-occurring disorders, concurrent disorders, co-existing disorders and dual disorders [5, 6]. Although language use may affect how the population is defined, the numbers show that this condition is widespread, as a Danish registry study demonstrated that the lifetime prevalence for substance use disorder was around 30% for all patients in contact with psychiatry [7]. Further, a Cochrane review implied that dual diagnosis is present in up to 75% of patients with severe mental illness [8]. More specifically, nearly half of all admitted male patients in psychiatry could be identified as having a dual diagnosis, although significant underreporting of dual diagnosis has been found [9].

The two conditions are complex, intertwined, and affect each other negatively, worsening treatment outcomes [10]. This challenges the treatment systems in many countries, which are often split into separate psychiatric- and substance use treatment [11, 12]. The literature of dual diagnosis often describes how patients cannot receive psychiatric treatment before their substance use disorder is treated and vice versa; thus when patients want to enter substance use treatment, they are told to stabilize their psychiatric illness first [13–15]. Reasons for this can be found in the difficult diagnostic process in practice, often requiring months of abstinence, and due to the persistent discussion of “*the chicken or the egg*” dilemma: is the psychiatric problem causing the substance use - or the opposite ? [13]. This places treatment responsibility in a grey zone, potentially leaving the patient inadequately treated. Further, it has been notoriously difficult for organizations to collaborate on this matter due to historical differences and positions in the power dynamics, leading to fragmented treatment [16].

Dual diagnosis treatment has traditionally been described as sequential, parallel, or integrated [6], meaning that the patient is treated for either: one condition at a time (sequential), both conditions simultaneously with different providers in different systems (parallel), or both conditions simultaneously within the same system by the same group of providers (integrated) [6]. In general, there is little evidence available regarding integrated treatment and its implicit non-pharmacological components [12, 17–20], often due to the low quality of studies in the field, risk of bias and few study participants [17]. Further, the most severe target groups, such as patients suffering from psychosis and substance use are often excluded [21]. However, integrated treatment as an ‘umbrella term’ is the recommended best practice in dual diagnosis treatment [5, 11], with one of the most studied and evidence-based approaches being Integrated Dual Diagnosis Treatment (IDDT) [22]. In Denmark, legislation stipulates that, from September 1st, 2024, integrated treatment should be provided in psychiatry for a minor subset of the most severe patients with DD (estimated 9200 individuals) [23]. This means that the vast majority of patients with moderate to severe dual diagnosis will *not* receive integrated treatment but will instead follow sequential or parallel treatment, in accordance with the existing procedures. This group of patients could therefore benefit from a *coordinated parallel treatment model*, which was developed in the current study to enhance coordination between the two separate systems, a solution that has been advocated in the literature [11, 24, 25], particularly when integrated treatment is neither a necessity nor an option. From a stepped care perspective [26, 27], this means that the least resource-demanding treatment is delivered initially, and the intensity can be turned up or down according to patient needs.

Over the years, there has been an increasing focus on studying the implementation of dual diagnosis treatment [28–30]. However, a recent report reviewing dual diagnosis guidelines from Denmark, Norway, Sweden, Great Britain, US, and Australia concluded that implementation still suffers from a lack of attention in the field [31, 32]. This analysis is further underlined in a review of the implementation of evidence-based practices in drug and alcohol treatment settings that concluded that implementation frameworks are generally unexploited [33], although some recent studies of DD *have* applied a framework in order to examine determinants for implementation [34–36]. Commonly, they have employed the Consolidated Framework for Implementation Research (CFIR), which is the most widely used determinant framework [37, 38]. Louie et al. have explored barriers and facilitators in relation to a new DD training program for staff [34], while Rollins et al. have focused on the implementation of Integrated Dual Diagnosis Treatment, IDDT [35]. Evans et al. have examined the organizational context of a collaborative care intervention for co‑occurring disorders through a survey aimed at clinicians in primary care [36]. To our knowledge, no studies have qualitatively examined cross-sector barriers and facilitators in relation to a dual diagnosis coordination model.

## Methods

### Study design

This study is an exploratory, qualitative study, using semi-structured interviews, with a focus on implementation determinants.

### Aim

The aim was to examine staff perceived barriers and facilitators to implementing a cross-sectoral coordination model for patients with dual diagnosis in order to use this knowledge in adapting and designing future coordinated treatment models and targeting implementation strategies.

### Setting

In 2007, a structural reform in Denmark delegated the responsibility for substance use treatment to the 98 municipalities, whereas psychiatric disorders belong under the jurisdiction of the hospital sector, governed by the five Regions of Denmark [39]. In 2016, it was decided that outpatient psychiatric treatment in the Capital Region of Denmark should be organized and delivered according to the Flexible Assertive Community Treatment model (FACT) [40]. While the original FACT model incorporates elements of Integrated Dual Diagnosis Treatment (IDDT) [40–42], which combined specialized substance use treatment with psychiatric treatment, this aspect has not been a primary focus in the regional implementation of FACT. Instead, the initial stages of implementation have emphasized areas such as FACT-board meetings, shared caseloads and outreach care [43].

The five municipal substance use treatment centers involved in this study, provide a broad range of psychosocial interventions and medical treatments (for reducing craving, withdrawal symptoms, detoxification, and substitution). The centers receive both walk-ins and referrals from a general practitioner, municipality, or hospital. Psychosocial interventions consist of, for example individual- and group therapy sessions (usually based on Cognitive Behavioral Therapy and/or Motivational Interviewing), social support and outreach visits. The staff at these facilities have various interdisciplinary backgrounds, and include doctors, nurses, social workers and pedagogues.

### The coordination model

**Fig. 1:**
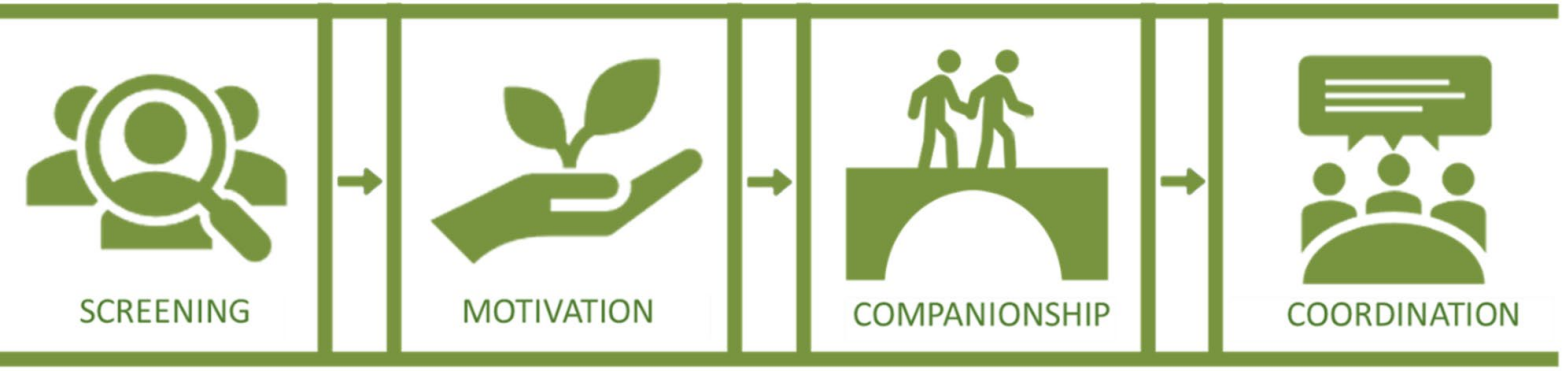
The SPOR Coordination Model

In 2021 the Capital Region of Denmark launched a Model Cell [44] to develop a treatment- and collaboration model for patients with dual diagnosis. This model aims at including a broad target group of patients with a substance use disorder combined with severe mental illness (SMI) - including but not limited to - psychosis and severe affective disorders causing low functional levels and a need for treatment in both sectors. In short, the Model Cell is a method to improve practice, by creating a small ‘test center’, where systematic processes of planning, testing, evaluation, and adaptation are performed in order to develop new concepts or improve existing ones [44]. The Coordination Model, SPOR [*da.* Sammenhængende Psykiatri- og Rusmiddelbehandling; or *Eng.* Coherent Psychiatry- and Substance use Treatment], is both the empirical object and the implementation object of this study (see Fig. 1).

Overall, the guiding principle of the coordination model is the ‘No wrong door’ approach, which means that, all points of entry to accessible services in public service systems are appropriate and services should provide direct linkage and coordination between them [45]. In this study, it means that no matter where the patient enters the system – whether it is a substance use treatment center or through psychiatry – rejection should be avoided and linkage provided. The model encompasses four components inspired by best practice and drawing on evidence-based practices within the field of dual diagnosis/substance use treatment providing a structured approach.

#### Screening

All patients in FACT are screened for problematic substance use with AUDIT and/or DUDIT [46, 47] at the initiation of treatment. The non-diagnostic screening is incorporated into the electronic patient journal (Epic^®^). In SUD treatment, the screening is done through conversations that draw on the Addiction Severity Index (ASI/EuropASI) [48, 49], tools which are not specialized for diagnostics of psychiatric disorders but can be used for mapping potential psychological and behavioral problems connected to the substance use.

#### Motivation

There is an enhanced focus on the motivational work required to encourage patients to enter treatment in the opposite sector, using methods well-known to staff: Motivational Interviewing (MI) [50] and/or Cognitive Behavioral Therapy (CBT) [51].

#### Companionship

With patient acceptance, the contact person in either psychiatry or substance use treatment follows the patient physically from A to B to initiate treatment in the opposite sector.

#### Coordination

At least three network meetings during a patient’s trajectory should be conducted, *and* before ending treatment the opposite sector must be informed. Network meetings involve a minimum of two professionals, preferably more, from different sectors or fields (e.g., hospital, community healthcare, job center, substance use treatment centers) plus the patient and possibly relatives meeting to establish and agree on a coherent plan for the patient [52].

Various implementation strategies were employed during the implementation process [53], preceding the results of this study. Implementation strategies are defined as “methods or techniques used to enhance the adoption, implementation, and sustainability of a clinical program or practice” [54]. These strategies represented a blend of planned actions and ad hoc measures selected by the project leader in response to the encountered challenges and expressed needs of participants. As part of the Model Cell method, this adaptive approach evolves dynamically in response to contextual barriers. For more details, please refer to Appendix 1, which provides a retrospective overview of the most frequently used implementation strategies categorized according to the Expert Recommendations for Implementing Change (ERIC) framework [53].

### Characteristics of participants and data collection

After agreement with managers from participating sites, potential informants were contacted in-person and via phone or mail regarding recruitment. The sampling was purposeful, striving for variation according to professional backgrounds and seniority [55], and participants should be acquainted with and/or have worked with the Coordination Model to some extent.

As shown in Appendix 2, participants represented a diverse range of professional backgrounds, including nurses, social workers, addiction counselors, psychologists, doctors, physiotherapists, occupational therapists, psychotherapists, and nursing assistants. The majority were nurses and social workers (*n* = 27). Nearly half of the participants had been employed in their current workplace for one year or less, while the longest tenure was 16 years. Total years in profession across participants ranged from 1 to 36 years (mean 13.8 years).

Interviews were conducted during the implementation phase between April and October 2023. To minimize interference in daily work, these were held in vacant offices at the participants’ respective workplaces. The total number of interviews was 24, involving 45 participants. Data material consisted of 16 individual interviews and 8 group interviews (ranging from 2 to 8 participants per group). Interviews were conducted with staff from six outpatient psychiatric hospital units (FACT teams), and from five municipal substance use treatment facilities, all located in the Capital Region of Denmark. All interviews were conducted by the first author (DMS). The duration of the interviews ranged from 35 to 91 min (mean 54 min). All interviews were audiotaped and transcribed verbatim.

The semi-structured interview guide (Appendix 3) was developed in collaboration with all authors on the basis of initial field observations by DMS and experienced researchers in the field of DD (SWD and KSJ) and implementation science (JWK), and guided by the five domains of the newly updated determinant framework *Consolidated Framework for Implementation Research* (CFIR) [37, 38].

### Analysis

The analytical process was conducted using *Qualitative Content Analysis* [56] and followed a series of systematic steps (examples are provided in Appendix 4). Initially, transcriptions were read thoroughly and repeatedly to gain an in-depth understanding of the data. Subsequently, the text was divided into meaning units. A condensation of the manifest content was then performed. Following this the units were deductively categorized into one of the five CFIR domains (1) Innovation, (2) Outer setting, (3) Inner setting, (4) Individual characteristics and (5) Implementation Process [38]. A column for latent interpretations was also included. Interpretations of the findings are presented in the Discussion section.

## Results

**Fig. 2:**
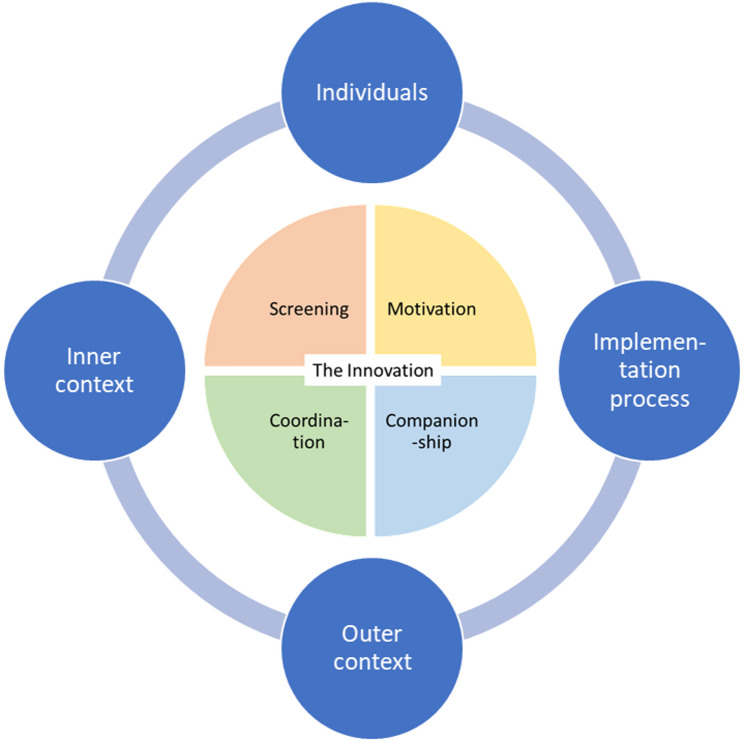
The SPOR model analyzed according to the five CFIR domains

The results are presented in the following sections, structured according to the five domains of the CFIR framework (Fig. 2). The *Innovation* domain consists of four parts (The SPOR model: screening, motivation, companionship, and coordination), which constitute the implementation object. This serves as the central focus, with the other domains interacting and affecting each other. To provide an overview of results, the main barriers and facilitators from all the domains are presented in Appendix 5.

### The innovation domain (the SPOR model)

The Coordination Model is the implementation object of this study, and its development and components are described in more detail in the Methods section. Overall, participants spoke positively about the model and its potential, expressing high hopes for its possibilities and attaching considerable value to its abilities. They found that it was not as comprehensive as other models they had tried earlier, and that the workload was not overwhelming. Others were a bit more skeptical or even disappointed since the model did not exceed all expectations.

#### Screening

Screening is the first component in SPOR. The implementation of alcohol and drug screening is progressing slowly but steadily in the FACT teams, vastly supported by its incorporation into patients’ electronic medical records. This way, the screening becomes an implicit part of available standard assessment schemes when receiving a patient. However, although screening is ideally conducted at the initial meeting several participants expressed concerns that this approach could jeopardize the therapeutic relationship.

*It’s a delicate balance to ask about drug use because many patients know from earlier experience*,* that revealing it can possibly exclude them from treatment. I wouldn’t necessarily bring it up at our first meeting*,* the relation needs to be established first (Int#11*,* FACT)*

The screening tool itself is scrutinized by staff, and the pros and cons are weighted. Most find it helpful because it is relatively quick and easy to fill out, and because it acts as a reminder to ask about alcohol, drugs and frequency of use. However, it is argued that it is also superficial and does not take the complexity of the substance use into account.

In substance use treatment centers, the focus is on detection of psychiatric illnesses among those patients in treatment for their substance use. Assessing psychiatric symptoms in dual diagnosis is considered very difficult by nearly all interview participants, who call for further education in this field, preferably by psychiatrists. Depending on experience, staff in both sectors use the screenings differently. Some use it systematically and just “*tick those boxes*”, others personalize it by adapting the questions to their own therapeutic conversation style.

#### Motivation

Motivating patients to enter treatment in the opposite sector, is the second component in SPOR. Nearly all participants agreed that this motivation process can be time-consuming and demanding for staff; however, perspectives varied on the fluctuating concept of motivation. Working with patient motivation in relation to the SPOR model requires adjustments at multiple levels, including changes in the embedded habits, question techniques, and therapeutic approaches of staff members. In FACT teams, for instance, a previous requirement of three months abstinence on the part of the client before initiating treatment and diagnostics had shaped past practices. With the introduction of the Coordination Model, this mindset is gradually shifting toward a harm reduction approach [57], where abstinence is no longer the sole criterion for treatment eligibility. This transition represents a significant cultural change for staff that requires long-term efforts. In contrast, harm reduction is already a more integrated practice in municipal substance use treatment centers. Yet even with this approach, participants in both sectors acknowledge that treatment is sometimes discontinued when patients fail to attend scheduled appointments.

*Well*,* if they don’t show up for their appointments*,* we’re quick to say: ”moving on*,* treatment terminated”. But it’s at exactly that time*,* they need extra support*,* ’cause there’s a reason why they don’t show up. It’s not that they aren’t motivated. Everybody that comes here is motivated*,* otherwise they wouldn’t be here (Int#7*,* SUD treat)*

Several participants expressed that violating system rules and not complying with agreements made can potentially label the patient as “not motivated”, even though the inability to show up can be a derivative symptom of either a psychiatric- or substance use disorder. Ultimately, labelling the patient as “not motivated” can be an excuse for not handling the problem in a system that lacks resources. Participants reflected on the inner motivation of the patients, since it determines their approach in their encounters with the patients, presenting a diverse spectrum ranging from believing that patients are 100% “controlled” by their addiction to believing that all patients are motivated.

It was further pointed out that it is important to know the practices, goals, and treatment offered by the opposite sector, as makes it easier both to motivate patients to accept treatment and to support patients during the treatment process.

#### Companionship

Companionship is the third component in the SPOR model. Physically accompanying patients to the opposite sector to initiate treatment is experienced as resource intensive but is overall spoken of in very positive terms and seen as a good investment, that strengthens collaboration and supports treatment compliance. The need for companionship is not necessarily limited to the first visit to the opposite sector, it may be beneficial to repeat this procedure several times to increase patients’ feeling of safety.

*We follow the patients and actually use quite a lot of resources to do it. But it gives us ‘plus points’ in the eyes of our clients […] it makes such great sense at so many levels […] Many of our clients have experienced multiple system failures*,* so good relations are truly significant to them. Us following them to the other sector is time really well spent (Int#3*,* SUD treat)*

Participants emphasized, however, the importance of clearly defined role distribution among staff during companionship visits to the opposite sector, also noting that the companionship has derivative effects for staff. It had enhanced their knowledge of each other to meet physically, see each other’s facilities, and experience first-hand how the patients’ problems are addressed there. Further, both sectors expressed the view that the SPOR coordination model enabled easier access to the opposite sector, especially from FACT teams to substance use centers. The reverse, from substance use centers to psychiatry, is generally still seen as slow and harder to plan and attain, as access to outpatient psychiatric treatment is referral-based despite increased collaboration.

#### Coordination

The fourth component of SPOR is coordination. While staff perceive network meetings as demanding in terms of time and resources, they also acknowledge their value in enhancing both treatment quality and cross-sector collaboration:

*At network meetings you gain a greater understanding for each other’s work processes. What is possible? What is not? Everything is brought to the table at once. Everyone has the same starting point with the client at the center (Int#11*,* SUD treat)*

These meetings provide an opportunity for staff to discuss complex cases and seek joint solutions and meet with strong acceptance among participants despite the required preparation and documentation. Moreover, many staff report that network meetings in general enhance patients’ sense of security and reassurance. Conversely, some also express concerns that these meetings may feel overwhelming for patients, which assigns considerable responsibility to staff to ensure that the meeting becomes a positive experience. During the implementation of the SPOR coordination model, the frequency of network meetings increased, yet still fell short of the target of at least three meetings per patient, highlighting an area for improvement. Coordination when ending treatment has shown to be particularly difficult to integrate into work processes:

*Coordination between sectors when patients end treatment is not going so well. We have had an enormous caseload*,* so focus has been on discharges. It is not my experience that we have a good routine for coordination when ending treatment yet(Int#8*,* FACT)*

This challenge was echoed by nearly all participants, who cited various reasons for the shortfall, including low prioritization, high workloads and practical obstacles.

Following this presentation of the Innovation domain, the next sections will present findings from the remaining four CFIR domains.

### Individuals domain

This section presents a synthesis of staff perspectives on patients with dual diagnosis (recipients of the innovation). The aim is to illustrate the heterogeneity and complexity of this patient population -characterized by severe mental illness and substance use disorders - from the standpoint of those providing care, since their perceptions directly influence their approach to the implementation of the SPOR model.

Overall, staff describe this patient group with a high degree of empathy, frequently using phrases such as, “*if it was me*…”, “*I would feel*…”, “*Imagine what they must feel*…” in an effort to understand patients’ experiences and consider how the system could be adapted to meet their needs. They emphasize that patients with DD have often faced multiple failures and hardships throughout their lives, from difficult childhoods to challenging adulthoods marked by adversity. Staff argue that encounters with the healthcare system should not serve as yet another obstacle, exemplified by the indignation expressed about the unequal treatment that this patient group experiences, when they struggle to maintain abstinence during treatment in psychiatry:

*A diabetic can have ice cream. A patient with lung disease is allowed to go smoke. Why can’t addicts be treated the same way?(Int#7*,* SUD treat)*

Replacing of stigmatizing words is a focus area in the SPOR model, which encourages the phrase ‘patient with substance use problem’ rather than ‘addict’ or ‘abuser’ [Danish word being *misbruger*]. However, despite these efforts, the term ‘addict’ remains prevalent in both sectors and as shown in this quote, even when staff express supportive intentions – here advocating equal treatment - unintentional stigmatization persists through the language used.

Further, patients with dual diagnosis are described by participants as having difficulties ‘fitting in’ and complying with system requirements, such as meeting times. Their lifestyle is frequently described as “*chaotic*”, which can cause non-compliance, although participants highlight that time spent on building relations and a high degree of staff flexibility are positive for the long-term treatment alliance. Staff recognize that the ways in which patients are approached and how help is offered can affect this alliance; therefore, the approach requires staff to exercise caution and empathy, since there is a risk for the patient if their capacities are challenged and they cannot live up to satff expectations. This potentially results in what staff have described as denial or dishonesty by patients, particularly in relation to substance use in terms of type, amount, and frequency.

### Inner context domain

Inner context describes the local circumstances of the implementing setting. Working with dual diagnosis is considered immensely difficult among participants in both sectors, and many describe the risk of de-prioritization in a busy practice. The target group is complex and requires a flexible approach that the participants struggle to sustain since time and resources are experienced as sparse. On the other hand, participants across both sectors have experienced a shift in internal culture over time. Repeated positive experiences have contributed to gradual behavior change in both attitudes and practices. For instance, the long-standing cultural practice of rejecting certain patients is slowly being dismantled:

*Now*,* we’re not allowed to reject anyone*,* which is overall a positive thing. But we fall short if a psychotic client enters. Then*,* we don’t know if we should correct his sense of reality or not. We don’t know![Int#5*,* SUD treat]*

Some express ambivalence regarding the changes in work processes introduced by SPOR, describing them as externally imposed; thus, they were regarded as top-down directives rather than internally motivated adaptations. A discrepancy between management and frontline practice levels is described, with certain initiatives perceived as non-negotiable, implemented without staff input.

Employees expressed a desire for increased managerial acknowledgement of staff effort during the individual patient trajectories. Although management is generally perceived as supportive at an overall level, the lack of visibility for SPOR-related activities in the formal data system creates a gap. Efforts such as going the ’extra mile’ for a patient, are described as largely ‘invisible’ to management. This lack of recognition may contribute to a perceived disconnect between frontline clinical realities and organizational reward structures, potentially undermining motivation and long-term engagement.

Non-attendance or sporadic appearance by patients is described in both sectors, which raises the question of when to end treatment, put the patient on ‘hold’ or keep the patient in active treatment contact. An attention point is the legal ‘treatment responsibility’. Can this be attained if the patient does not show up? Working with the most severe patients is described by some as professionally unrewarding.

*The ones with the most severe abuse are the hardest ones to collaborate with. Their abuse overshadows almost everything*,* so we can’t treat them. It is totally unsatisfying when they are connected to the outpatient clinic because we actually can’t work with them at all. Their lives are so chaotic*,* and they take up a lot of staff resources. [Int#4*,* group*,* FACT]*

This quote reflects a broader sense of professional powerlessness. When staff feel that their clinical effort have little or no impact it may foster frustration and emotional fatigue.

Some question whether *all* employees should work with dual diagnosis and suggest a reorganization so that only those with a special interest in the topic should work in this area. On the other hand, some argue that it must be a responsibility for all since the majority of patients in psychiatry are known to have a substance use problem.

### Outer context domain

This domain describes how participants from the two sectors perceive each other’s work in regard to dual diagnosis patients. Commonly, they point towards the “double work” done in dual diagnosis treatment, and how they have come to realize this, through the implementation of the SPOR model:

*Many of the problems they talk about [in substance use treatment] are exactly the same as we talk about here [in psychiatry]. Everyday life*,* lack of structure*,* desires and goals. Many patients will probably find that conversations in the two sectors have the same content”(Int#11*,* FACT)*

Although both sectors illuminate what unifies them and call for more knowledge of this, they also present opposing stances according to sector. Staff in the substance use centers mentioned several times that separating practices in psychiatric outpatient clinics from in-hospital wards is challenging for outsiders; hence, the encounter with “psychiatry” as a whole is to some extent perceived as inflexibly rigid. Further, it is highlighted that approaches to the patients in substance use treatment and psychiatry differ – presenting harm reduction with long perspectives as opposed to flow and diagnostics.

While knowledge has improved with SPOR, a lack of knowledge of each other’s competencies and treatment offers still exists and leads to frustration among some participants:

*Who are they? What do they look like? What do they do? What options do they have? I have no clue! […] For me it’s like… I know it’s there [the treatment center]*,* but I don’t know what it is*,* how it looks and who works there (Int#4*,* group*,* FACT)*

Participants from both sectors emphasize the accessibility of their counterpart, describing how communication has clearly improved with SPOR; however, several still feel that it mainly occurs on their own initiative, and observe how difficult it is to get in touch with staff in the other sector – in substance use treatment due to opening times and telephone hours, and in psychiatry due to high staff turnover. Further, both sectors criticize the other for ending treatment too soon, since they are unable to deliver suitable treatment, or the patient is considered too difficult for them to handle.

### The implementation process domain

This domain showcases how participants (deliverers of the intervention) perceived the implementation process and strategies, as well as their own personal needs and requests for future implementation efforts. The majority of participants mention that they need more knowledge about dual diagnosis, as well as ongoing education to sustain the knowledge they have already acquired. They find it hard to communicate the rationale for coordinated treatment to service users convincingly when their knowledge is neither complete nor updated. More specifically, staff in SUD treatment call for more knowledge on psychiatric diagnostic criteria and how different diagnoses are expressed clinically. Psychiatry staff, on the other hand, call for knowledge about substance use criteria and how to address the use.

As shown in Appendix 1 (implementation strategies), numerous types of meetings have been held as part of the implementation process. While most participants did not problematize the frequency of meetings, some initially perceived them as excessive, although later acknowledged their impact:

*Then they [project management team] arrived at our morning meetings once a week and they were like: “What’s up? What’s happened since last time?” – and not a damn thing had happened! It doesn’t go that fast! It became… an annoyance and a pressure. But then*,* eventually we were all proved wrong*,* it just took some time(Int#5*,* FACT)*

Further, all participants highlighted the importance of repetition, reminders, and practical tools to support implementation calling for action cards and guidelines, as well as posters with contact e-mails, phone numbers, target groups, and specific treatment offers at the different sites. While some of these materials had already been introduced, participants stressed the need for further concretization and visibility to support execution. Several people in this project have acted as facilitators; most often mentioned is a cross-sector secretary handling the booking of network meetings (see Appendix 4) and the project management team. These actors demonstrated championing behavior characterized by enthusiasm, support, and trust building. Although it is an admission of fragility to display internal problems, lack of knowledge, and insecurities in cross-sector collaboration, participants highlighted the psychological safety fostered by the project management team, which enabled staff to express uncertainty and knowledge gaps - an important condition for sustained learning and cross-sector collaboration. Nonetheless, several participants noted that cultural changes take time and emphasized that SPOR elements had not yet become fully integrated into daily routines, although they were moving toward it. Other important facilitators highlighted by staff were the managers, who were mainly described as supportive and encouraging. Overall, employees have felt very involved in the entire design- and implementation process; however, some also expressed concern about a recent perceived shift in the implementation process—from involvement to increased top-down steering:

*Now it has been moved to an executive level*,* where only managers meet. And I’ve got a feeling that the rest of us are left behind. There is some information we don’t receive*,* and we’re not updated like we used to be – and that is important if we need everybody to do this* *(Int#4*,* SUD treat)*

Regarding future implementation efforts, participants across both sectors present similar suggestions. Crucially, they advised against premature scaling of SPOR until existing implementation barriers were fully addressed. They also cautioned against the risk of implementation fatigue due to competing initiatives in clinical practice. To reach a higher degree of implementation and obtain sustainability, participants offered a number of proposals: structured onboarding for new staff, reinforcement of the SPOR narrative, prioritization of physical meetings (e.g. companionship and network meetings) and development of guidelines with concrete “how-to” instructions. Finally, several suggest a shared cross-sector conference forum, potentially online, where difficult generalized cases can be discussed with the aim of fostering mutual new insights and future joint solutions.

## Discussion

By applying the Consolidated Framework for Implementation Research (CFIR), this study has aimed to showcase the barriers and facilitators perceived by frontline staff to a cross-sectoral coordination model for dual diagnosis [38]. The CFIR-guided analysis displayed nuanced perspectives from both psychiatric outpatient clinics and substance use treatment services. While some perspectives were shared across sectors, others diverged (Appendix 5). In the following section, we discuss and interpret the key findings, before framing them by a forward-looking implementation perspective.

A consistent theme emerging across the CFIR domains is that the frontline – whether it is in psychiatry or the municipality – is the place where structural problems unfold and need problem-solving in the direct encounter with the opposite sector. *Street-level bureaucracy* is a concept coined by Michael Lipsky to explain the routine work practices involved in public workers’ encounters with clients at the frontline of the welfare system [58]. Here, they act as both implementers and interpreters of policy in their routine interactions with clients. The findings suggest that although participants in each sector often operate from different structural positions, they frequently share similar perspectives on coordination challenges. This is evident in both the *inner*- and *outer context* CFIR domains, as staff in both sectors struggle with sparse resources and increasing performance demands. At the sector ‘border’, these pressures are expressed as mutual gatekeeping, with each side perceiving the other as prematurely ending treatment or refusing services. A component in SPOR is that patients should not be rejected, which is a considerable change in practice for both sectors. Yet, as the results show, both sectors continue to accuse the other of rejecting clients or ending treatment too soon. According to Lipsky, different types of strategies are conducted to ‘help’ frontline workers handle system pressure and rationing of services is a way to manage resource constraints [58]. In this study, decisions to terminate care were often justified by citing patients’ lack of motivation or irregular attendance. This reflects a form of “*creaming”*, whereby services are directed toward those deemed more likely to benefit, while others are deprioritized [58]. Frontline workers from both sectors are thus constantly left with the responsibility for solving profound problems of structural dimensions with limited room for case-by-case maneuver. Several participants pointed out that during implementation of SPOR it occurred to them that duplication and overlap of methods and tasks were present when working with dual diagnosis patients. This recognition of a common ground holds the potential for fostering closer collaborations; however, the focus often remains fixated on sectoral differences rather than commonalities. Further, a fundamental power discrepancy has been described in the field of dual diagnosis in Denmark, where the power balance is uneven, since there is free access to municipal treatment, whereas psychiatry is referral based [16].

One of the primary facilitators identified in our material was that staff in both sectors generally perceived the coordination model as a *relative advantage* compared to prior limited cross-sector collaboration. According to Rogers’ classic *diffusion of innovations* implementation theory, which is also partly incorporated into CFIR constructs, five attributes influence the adoption of an innovation: relative advantage, compatibility, complexity, trialability and observability [59]. *Relative advantage* was a prominent theme in this study, as staff frequently spoke positively about the model’s potential and felt involved by the project management team in the process of designing and developing it. The second attribute, *compatibility [cf. Rogers]*, also appeared to be satisfied to some degree, since the model was co-designed with staff, it was generally perceived to align with their values, needs, and experiences. The implementation process also supported a high degree of *trialability* [cf. Rogers], since participants were able to suggest adjustments according to Model Cell-thinking, as described in the methods section. Furthermore, continuous support and facilitation from the project management team and prioritization by leadership were highlighted as critical factors for successful implementation. These findings align with those of Louie et al., who developed a dual diagnosis training program for staff, identifying self-efficacy, engaged leadership, and active clinical champions as key facilitators [34].

Nevertheless, several barriers were identified in the material (see Appendix 5). Prominent among these were perceptions of a lack of training, lack of knowledge, limited time, and resources. These findings mirror previous studies of dual diagnosis examining integrated dual diagnosis treatment rather than coordinated treatment [28, 35, 36]. Although, the coordination model might seem rather simple and not verycomprehensive, participants reported that its four components involve procedural changes and behavioral shifts that introduce *complexity* [cf. Rogers]. From an implementation science perspective, entrenched habits function as ‘cognitive shortcuts’ and breaking these require contextual cues [e.g. reminders] or the intrinsic motivation for doing so [60]. Reminders were consistently used as a strategy in the implementation phase (Appendix 1), and included verbal prompts (e.g. meetings), physical artifacts (e.g., posters, flyers, actions cards etc.) and electronic tools (e.g. screening available in patients’ journals). Despite these strategies, participants emphasized a continued need for concrete practical knowledge in the form of, extensive reminders, information, educational visits etc., if SPOR should become a part of routine practice for all employees. This is in line with the findings of Rollins et al., who also used CFIR as an analytic framework, when they describe how participants called for very concrete hands-on help in how to execute the different steps in practice [35].

Two of the major components in SPOR are network meetings and companionship, which received both positive and negative feedback from participants for being concrete and action-oriented. The potential of network meetings was highlighted several times as bringing cross sectoral co-workers together, gaining an understanding of each other. Yet, it was also brought to attention that this type of meeting is demanding, requiring planning, coordination, preparation of the patient, clarification of roles and so on. Similar barriers are documented in other Danish studies focusing on network meetings in relation to recovery from psychiatric disorders, suggesting the need for a structured and clear agenda, patient focus, and clarity in agreements [61]. Additionally, the companionship element involving the co-presence of staff across sectors, was generally well received and seen as a concrete embodiment of cross-sector collaboration. However, a key implementation barrier was the lack of formal allocation of time and resources for this activity. Although management supported the overall model, the absence of registration options or designated time undermined its feasibility. In terms of Rogers’ *observability* [ie. is it possible for others to see what you are doing?], this lack of visibility and acknowledgement in organizational systems can hinder adoption, as employees may feel their efforts are unsupported or unrecognized.

Implementation science has increasingly sought to link specific barriers to targeted strategies, using tools like conjoint analysis and intervention mapping [62]. However, there is no definitive formula for selecting optimal strategies. In this study, numerous barriers existed despite intensive efforts by the project management team to address them with planned, multifaceted, ad hoc implementation strategies (Appendix 1). Savic et al. [63] conducted a systematic review of strategies for implementing integrated care for individuals with alcohol and substance use disorders in both primary and secondary care, highlighting the use of multilevel strategies across funding, organizational, service delivery, and clinical domains [63]. While this study concerns coordinated rather than integrated care, many of these strategies are transferable. Based on our findings, future implementation efforts for the SPOR model may benefit from enhancing strategies related to: *common goals*, *information sharing*, *professional networks*, and *joint care planning* [63]. Moreover, recent studies have reported on the successful application of NIATx strategies, a multifaceted approach to implementing integrated dual diagnosis treatment [64–66]. In an RCT, organizations applying NIATx strategies significantly improved their capacity to deliver integrated care services for dual diagnosis [64]. A comparison of strategies in NIATx (e.g. identifying champions, local consensus discussions, educational outreach and materials) [66] with those used in SPOR (Appendix 1), reveals a considerable overlap. This suggests that, despite variations in dosage and frequency, these combined strategies hold promise for the effective and sustainable implementation of dual diagnosis services.

### Strengths and limitations

This study has several notable strengths. First, it presents a cross-sectoral collaborative model for managing dual diagnosis, which contributes to the sparse literature in this field. Second, data were collected from two sectors and findings were reported in accordance with the consolidated criteria for reporting qualitative research (COREQ) [67], which enhances transparency and strengthens the study’s credibility. Third, the study includes a broad and diverse sample of participants with varying professional backgrounds reflecting everyday practice, which increases the transferability of findings to real-world practice. Fourth, the use of the CFIR framework provided a structured and theoretical analytic approach, Finally, the study not only explores barriers and facilitators but also highlights implementation strategies and points towards future efforts, adding practical relevance.

However, some limitations are also present. In relation to sampling strategy, we cannot know if those who chose to participate in interviews were generally more positive than those not participating. However, since numerous barriers and negative opinions were also expressed, we believe that the sampling was sufficiently broad. Dynamics in group interviews may have influenced the outcome, since it is well-known that dominating colleagues can set the course of a group discussion. Nevertheless, a group interview also has the potential to test participants’ opinions in a way that does not occur in single interviews, since colleagues can oppose a statement and correct each other if there is a mismatch between what they say they do, and what they actually do [68].

Further, this article provides an instant picture of barriers and facilitators, although it has been found that barriers may change over time [69]. By applying a well-known framework such as CFIR [38] the study establishes a foundation for future follow-up research and enables comparison with other CFIR- based studies in the field of dual diagnosis. The comprehensiveness of CFIR is a double-edged sword: while it offers an extensive analytical lens, its many sub-constructs pose challenges for consistency and replicability across studies. To address this, we decided to present findings at the domain level to facilitate comparability, while acknowledging that some analytical nuances may be lost in this approach.

## Conclusion

Did the SPOR coordination model succeed in bridging the gap between the two sectors and clarifying patient pathways? The answer is not straightforward. Frontline staff experienced both barriers and facilitators across all five CFIR domains, reflecting the complex and dynamic nature of implementation in a real-world setting.

This study contributed to a heightened awareness of the importance of screening and diagnostics procedures, recognized the value of motivational efforts, promoted harm reduction strategies, and fostered improved cross-sectoral relationships through joint activities such as network meetings and coordinated efforts in clinical practice. Still, persistent challenges were also identified, including cultural and behavioral differences between sectors, and gaps in competences, knowledge, and resources. These findings highlight the importance of designing or selecting future implementation strategies that explicitly address these barriers. Efforts should focus on capacity-building, mutual training across sectors, sustained leadership engagement, and long-term resource allocation to ensure that positive developments are maintained and expanded. Strengthening trust, clarifying roles, and aligning expectations between sectors will be essential in order to establish durable and effective cross-sectoral collaboration in dual diagnosis care.

## Supplementary Information

Below is the link to the electronic supplementary material.

Supplementary Material 1

Supplementary Material 2

Supplementary Material 3

Supplementary Material 4

Supplementary Material 5

## Data Availability

No datasets were generated or analysed during the current study.

## Abbreviations

ASI: Addiction Severity Index
AUDIT: Alcohol Use Disorder Identification Test
CBT: Cognitive Behavioral Therapy
CFIR: Consolidated Framework for Implementation Research
DD: Dual Diagnosis
DUDIT: Drug Use Disorder Identification Test
ERIC: Expert Recommendations for Implementing Change
FACT: Flexible Assertive Community Treatment
IDDT: Integrated Dual Diagnosis Treatment
MI: Motivational Interviewing
SPOR: [da. Sammenhængende Psykiatri og Rusmiddelbehandling], eng. Coherent Psychiatry and Substance Use Treatment
SUD: Substance Use Disorder

## References

[1] Batki SL, Meszaros ZS, Strutynski K, Dimmock JA, Leontieva L, Ploutz-Snyder R, et al. Medical comorbidity in patients with schizophrenia and alcohol dependence. Schizophr Res. 2009;107(2–3):139–46. 10.1016/j.schres.2008.10.016. 19022627 10.1016/j.schres.2008.10.016 PMC2649875

[2] Momen NC, Plana-Ripoll O, Agerbo E, Benros ME, Børglum AD, Christensen MK, et al. Association between Mental Disorders and Subsequent Medical Conditions. N Engl J Med. 2020;382(18):1721–31. 10.1056/NEJMoa1915784. 32348643 10.1056/NEJMoa1915784 PMC7261506

[3] Ness O, Borg M, Davidson L. Facilitators and barriers in dual recovery: a literature review of first-person perspectives. Adv Dual Diagnosis. 2014;7(3):107–17. 10.1108/ADD-02-2014-0007.

[4] World Health Organization. Lexicon of Alcohol and Drug Terms. Geneva (CH): World Health Organization; 1994.

[5] Hakobyan S, Vazirian S, Lee-Cheong S, Krausz M, Honer WG, Schutz CG. Concurrent disorder management guidelines: systematic review. J Clin Med. 2020;9(8). 10.3390/jcm9082406. 10.3390/jcm9082406 PMC7463987 32731398

[6] Substance Abuse and Mental Health Services Administration. Substance Use Disorder Treatment for People With Co-Occurring Disorders: Updated 2020. Treatment Improvement Protocol (TIP) Series. Rockville (MD): Substance Abuse and Mental Health Services Administration (US); 2020. 34106563

[7] Toftdahl NG, Nordentoft M, Hjorthøj C. Prevalence of substance use disorders in psychiatric patients: a nationwide Danish population-based study. Soc Psychiatry Psychiatr Epidemiol. 2016;51(1):129–40. 10.1007/s00127-015-1104-4. 26260950 10.1007/s00127-015-1104-4

[8] Temmingh HS, Williams T, Siegfried N, Stein DJ. Risperidone versus other antipsychotics for people with severe mental illness and co-occurring substance misuse. Cochrane Database Syst Rev. 2018;1(1):Cd011057. 10.1002/14651858.CD011057.pub2. 29355909 10.1002/14651858.CD011057.pub2 PMC6491096

[9] Mårtensson S, Düring SW, Johansen KS, Tranberg K, Nordentoft M. Time trends in co-occurring substance use and psychiatric illness (dual diagnosis) from 2000 to 2017 - a nationwide study of Danish register data. Nord J Psychiatry. 2023;77(4):411–9. 10.1080/08039488.2022.2134921. 36271867 10.1080/08039488.2022.2134921

[10] Antai-Otong D, Theis K, Patrick DD. Dual Diagnosis: Coexisting Substance Use Disorders and Psychiatric Disorders. Nurs Clin North Am. 2016;51(2):237–47. 10.1016/j.cnur.2016.01.007. 27229278 10.1016/j.cnur.2016.01.007

[11] Fantuzzi C, Mezzina R. Dual diagnosis: A systematic review of the organization of community health services. Int J Soc Psychiatry. 2020;66(3):300–10. 10.1177/0020764019899975. 31957528 10.1177/0020764019899975

[12] Torrens M, Rossi PC, Martinez-Riera R, Martinez-Sanvisens D, Bulbena A. Psychiatric co-morbidity and substance use disorders: treatment in parallel systems or in one integrated system? Subst Use Misuse. 2012;47(8–9):1005–14. 10.3109/10826084.2012.663296. 22676568 10.3109/10826084.2012.663296

[13] Groenkjaer M, de Crespigny C, Liu D, Moss J, Cairney I, Lee D, et al. The Chicken or the Egg: Barriers and Facilitators to Collaborative Care for People With Comorbidity in a Metropolitan Region of South Australia. Issues Ment Health Nurs. 2017;38(1):18–24. 10.1080/01612840.2016.1233596. 27740880 10.1080/01612840.2016.1233596

[14] Johansen KS. Dobbelt diagnose - Dobbelt behandling. Glostrup: KABS VIDEN; 2009.

[15] Merinder LB. Psykiatriens Stedbørn. STOF Tidsskrift Stofmisbrugsområdet. 2007;9:28–9.

[16] Johansen KS. Models for Cooperation and Positions of Power. Qualitative Stud. 2018;5(2):125–39.

[17] Hunt GE, Siegfried N, Morley K, Brooke-Sumner C, Cleary M. Psychosocial interventions for people with both severe mental illness and substance misuse. Cochrane Database Syst Rev. 2019;12(12):CD001088. 10.1002/14651858.CD001088.pub4. 31829430 10.1002/14651858.CD001088.pub4 PMC6906736

[18] TietQQ, Mausbach B. Treatments for patients with dual diagnosis: A review. Alcohol Clin Exp Res. 2007;31(4):513-36. 10.1111/j.1530-0277.2007.00336.x 10.1111/j.1530-0277.2007.00336.x 17374031

[19] Kikkert M, Goudriaan A, de Waal M, Peen J, Dekker J. Effectiveness of Integrated Dual Diagnosis Treatment (IDDT) in severe mental illness outpatients with a co-occurring substance use disorder. J Subst Abuse Treat. 2018;95:35–42. 10.1016/j.jsat.2018.09.005. 30352668 10.1016/j.jsat.2018.09.005

[20] Siddiqui S, Mehta D, Coles A, Selby P, Solmi M, Castle D. Psychosocial Interventions for Individuals With Comorbid Psychosis and Substance Use Disorders: Systematic Review and Meta-analysis of Randomized Studies. Schizophr Bull. 2024. 10.1093/schbul/sbae101. 38938221 10.1093/schbul/sbae101

[21] Düring SW, Sivertsen DM, Johansen KS. Non-Pharmacological Components in Integrated Treatment for Patients with Dual Diagnosis: A Scoping Review. J Dual Diagn. 2025;21(2):120–41. 10.1080/15504263.2025.2478900. 40112128 10.1080/15504263.2025.2478900

[22] Mueser N. Drake, Smith. Integrated Treatment for Dual Disorders: A Guide to Effective Practice. New York: Guilford; 2003.

[23] Danish Ministry of Finance. Aftale om Regionernes økonomi for 2024. Copenhagen: Danish Ministry of Finance; May 2023. https://fm.dk/media/xdqltojf/aftale-om-regionernes-oekonomi-for-2024-a.pdf [.

[24] Vitali M, Sorbo F, Mistretta M, Coriale G, Messina MP, Alessandrini G, et al. Drafting a dual diagnosis program: a tailored intervention for patients with complex clinical needs. Riv Psichiatr. 2018;53(3):149–53. 10.1708/2925.29417. 29912217 10.1708/2925.29417

[25] Heath RW, Washington, D.C.: SAMHSA-HRSA Center for Integrated Health Solutions; 2013.

[26] Bower P, Gilbody S. Stepped care in psychological therapies: access, effectiveness and efficiency. Narrative literature review. Br J Psychiatry. 2005;186:11–7. 10.1192/bjp.186.1.11. 15630118 10.1192/bjp.186.1.11

[27] Cross SP, Hickie I. Transdiagnostic stepped care in mental health. Public Health Research & Practice. 2017;27(2). 10.17061/phrp2721712. 10.17061/phrp2721712 28474049

[28] Brunette MF, Asher D, Whitley R, Lutz WJ, Wieder BL, Jones AM, et al. Implementation of integrated dual disorders treatment: a qualitative analysis of facilitators and barriers. Psychiatr Serv. 2008;59(9):989–95. 10.1176/ps.2008.59.9.989. 18757591 10.1176/ps.2008.59.9.989

[29] Sylvain L. Studying Implementation of Dual Diagnosis Services: A Review. J Dual Diagnosis. 2013;9(2):195–207. 10.1080/15504263.2013.771809.

[30] Damschroder LJ, Hagedorn HJ. A guiding framework and approach for implementation research in substance use disorders treatment. Psychol Addict Behav. 2011;25(2):194–205. 10.1037/a0022284. 21443291 10.1037/a0022284

[31] Thylstrup J, Schrøder J. Unge og dobbeltdiagnose (1): Rapport om retningslinjer om udredning og behandling uden for Danmark. Aarhus (DK): Center for Rusmiddelforskning, Aarhus Universitet and Kompetencecenter for Dobbeltdiagnoser, Region Hovedstadens Psykiatri; 2023b.

[32] Thylstrup J, Schrøder J, Johansen J. Unge og dobbeltdiagnose (3): Rapport om udarbejdning og implementering af udenlandske og danske retningslinjer om udredning og behandling. Aarhus (DK): Center for Rusmiddelforskning, Aarhus Universitet and Kompetencecenter for Dobbeltdiagnoser, Region Hovedstadens Psykiatri; 2023.

[33] Louie E, Barrett EL, Baillie A, Haber P, Morley KC. A systematic review of evidence-based practice implementation in drug and alcohol settings: applying the consolidated framework for implementation research framework. Implement Sci. 2021;16(1):22. 10.1186/s13012-021-01090-7. 33663523 10.1186/s13012-021-01090-7 PMC7931583

[34] Louie EGV, Baillie A, Uribe G, Wood K, Teesson M, Childs S, Rogers D, Haber PS, Morley KC. Barriers and Facilitators to the Implementation of the Pathways to Comorbidity Care (PCC) Training Package for the Management of Comorbid Mental Disorders in Drug and Alcohol Settings. Front Health Serv. 2021;1:785391. 10.3389/frhs.2021.785391. 36926478 10.3389/frhs.2021.785391 PMC10012778

[35] Rollins ALEJ, Kukla M, Wasmuth S, Salyers MP, McGuire AB. Implementation of Integrated Dual Disorder Treatment in Routine Veterans Health Administration Settings. Int J Mental Health Addict. 2024;22:578–98. 10.1007/s11469-022-00891-1. 10.1007/s11469-022-00891-1 PMC13245570 42266573

[36] Evans SK, Dopp A, Meredith LS, Ober AJ, Osilla KC, Komaromy M, et al. Findings from an Organizational Context Survey to Inform the Implementation of a Collaborative Care Study for Co-occurring Disorders. J Behav Health Serv Res. 2024;51(1):4–21. 10.1007/s11414-023-09851-6. 37537428 10.1007/s11414-023-09851-6 PMC10733218

[37] Damschroder LJ, Aron DC, Keith RE, Kirsh SR, Alexander JA, Lowery JC. Fostering implementation of health services research findings into practice: a consolidated framework for advancing implementation science. Implement Sci. 2009;4:50. 10.1186/1748-5908-4-50. 19664226 10.1186/1748-5908-4-50 PMC2736161

[38] Damschroder LJ, Reardon CM, Widerquist MAO, Lowery J. The updated Consolidated Framework for Implementation Research based on user feedback. Implement Sci. 2022;17(1):75. 10.1186/s13012-022-01245-0. 36309746 10.1186/s13012-022-01245-0 PMC9617234

[39] Christiansen T. Ten years of structural reforms in Danish healthcare. Health Policy. 2012;106(2):114–9. 10.1016/j.healthpol.2012.03.019. 22521580 10.1016/j.healthpol.2012.03.019

[40] van Veldhuizen JR. FACT: a Dutch version of ACT. Community Ment Health J. 2007;43(4):421–33. 10.1007/s10597-007-9089-4. 17514502 10.1007/s10597-007-9089-4

[41] Mueser KT, Drake RE, Noordsy DL, Smith PR. Integrated treatment for dual disorders: A guide to effective practice. New York (NY): Guilford Press; 2003.

[42] Smith D, Mueser, Brunette B. McGovern, et al. Integrated Dual Disorders Treatment. Best Practices, Skills, and Resources for Successful Client Care. Dartmouth Psychiatric Research Center: Hazelden Publishing; 2010.

[43] Nielsen CM, Hjorthøj C, Nordentoft M, Christensen U. A Qualitative Study on the Implementation of Flexible Assertive Community Treatment - an Integrated Community-based Treatment Model for Patients with Severe Mental Illness. Int J Integr Care. 2021;21(2):13. 10.5334/ijic.5540. 33981190 10.5334/ijic.5540 PMC8086721

[44] Toussaint A. Management on the Mend: The Healthcare Executive Guide to System Transformation. Catalysis; 2015.

[45] South Western Sydney Local Health District. No Wrong Door. Available from: https://nowrongdoor.org.au/mental-health-charter/

[46] Babor T, Biddle-Higgins J, Saunders J, Monteiro M. The alcohol use disorders identification test: Guidelines for use in primary care. Geneva, Switzerland: World Health Organization; 2001.

[47] Berman AH, Bergman H, Palmstierna T, Schlyter F. Evaluation of the Drug Use Disorders Identification Test (DUDIT) in criminal justice and detoxification settings and in a Swedish population sample. Eur Addict Res. 2005;11(1):22–31. 10.1159/000081413. 15608468 10.1159/000081413

[48] McLellan AT, Kushner H, Metzger D, Peters R, Smith I, Grissom G, et al. The Fifth Edition of the Addiction Severity Index. J Subst Abuse Treat. 1992;9(3):199–213. 10.1016/0740-5472(92)90062-s. 1334156 10.1016/0740-5472(92)90062-s

[49] Scheurich A, Müller MJ, Wetzel H, Anghelescu I, Klawe C, Ruppe A, et al. Reliability and validity of the German version of the European Addiction Severity Index (EuropASI). J Stud Alcohol. 2000;61(6):916–9. 10.15288/jsa.2000.61.916. 11188499 10.15288/jsa.2000.61.916

[50] Miller WR. Motivational Interviewing: Helping People Change and Grow. 4th ed. New York (NY): Guilford Press; 2023.

[51] Beck JS. Cognitive Therapy: Basics and Beyond. New York: Guildford; 1964.

[52] Mental Health Services. Netvaerksmøde skabelon (Network meeting template). 2018 [Available from: https://www.psykiatri-regionh.dk/netværksmøder

[53] Powell BJ, Waltz TJ, Chinman MJ, Damschroder LJ, Smith JL, Matthieu MM, et al. A refined compilation of implementation strategies: results from the Expert Recommendations for Implementing Change (ERIC) project. Implement Sci. 2015;10:21. 10.1186/s13012-015-0209-1. 25889199 10.1186/s13012-015-0209-1 PMC4328074

[54] Proctor P. McMillen. Implementation strategies: recommendations for specifying and reporting. Implement Sci. 2013;8:139. 24289295 10.1186/1748-5908-8-139 PMC3882890

[55] Green J, Thorogood N. Qualitative Methods for Health Research. 2nd ed. London (UK): SAGE Publications; 2009.

[56] Graneheim UH, Lundman B. Qualitative content analysis in nursing research: concepts, procedures and measures to achieve trustworthiness. Nurse Educ Today. 2004;24(2):105–12. 10.1016/j.nedt.2003.10.001. 14769454 10.1016/j.nedt.2003.10.001

[57] Riley D, Pates R. Harm Reduction in Substance Use and High-Risk Behaviour: International Policy and Practice. West Sussex: Wiley-Blackwell; 2012.

[58] Lipsky M, Street-Level B. Dilemmas of the individual in public services. New York: Russell Sage Foundation; 2010.

[59] Rogers EM. Diffusion of innovations. 5th ed. New York, NY: Free; 2003.

[60] Nilsen P, Roback K, Broström A, Ellström PE. Creatures of habit: accounting for the role of habit in implementation research on clinical behaviour change. Implement Sci. 2012;7:53. 10.1186/1748-5908-7-53. 22682656 10.1186/1748-5908-7-53 PMC3464791

[61] Jørgensen K, Rasmussen T, Hansen M, Andreasson K, Karlsson B. Recovery-Oriented Network Meetings in Mental Healthcare: A Qualitative Study. Issues Ment Health Nurs. 2022;43(2):164–71. 10.1080/01612840.2021.1961178. 34469284 10.1080/01612840.2021.1961178

[62] Powell BJ, Beidas RS, Lewis CC, Aarons GA, McMillen JC, Proctor EK, et al. Methods to Improve the Selection and Tailoring of Implementation Strategies. J Behav Health Serv Res. 2017;44(2):177–94. 10.1007/s11414-015-9475-6. 26289563 10.1007/s11414-015-9475-6 PMC4761530

[63] Savic M, Best D, Manning V, Lubman DI. Strategies to facilitate integrated care for people with alcohol and other drug problems: a systematic review. Subst Abuse Treat Prev Policy. 2017;12(1):19. 10.1186/s13011-017-0104-7. 28388954 10.1186/s13011-017-0104-7 PMC5384147

[64] Assefa MT, Ford JH 2nd, Osborne E, McIlvaine A, King A, Campbell K, et al. Implementing integrated services in routine behavioral health care: primary outcomes from a cluster randomized controlled trial. BMC Health Serv Res. 2019;19(1):749. 10.1186/s12913-019-4624-x. 10.1186/s12913-019-4624-x PMC6814122 31651302

[65] Chokron Garneau H, Assefa MT, Jo B, Ford JH 2nd, Saldana L, McGovern MP. Sustainment of Integrated Care in Addiction Treatment Settings: Primary Outcomes From a Cluster-Randomized Controlled Trial. Psychiatr Serv. 2022;73(3):280–6. 10.1176/appi.ps.202000293. 10.1176/appi.ps.202000293 PMC8814048 34346729

[66] Ford JH 2nd, Zehner ME, Schaper H, Saldana L. Adapting the stages of implementation completion to an evidence-based implementation strategy: The development of the NIATx stages of implementation completion. Implement Res Pract. 2023;4:26334895231200379. 10.1177/26334895231200379. 10.1177/26334895231200379 PMC10510360 37790170

[67] Tong A, Sainsbury P, Craig J. Consolidated criteria for reporting qualitative research (COREQ): a 32-item checklist for interviews and focus groups. Int J Qual Health Care. 2007;19(6):349–57. 17872937 10.1093/intqhc/mzm042

[68] Kitzinger J. Introducing focus groups. BMJ. 1995;311:299–302. 7633241 10.1136/bmj.311.7000.299 PMC2550365

[69] Petersen HV, Sivertsen DM, Jørgensen LM, Petersen J, Kirk JW. From expected to actual barriers and facilitators when implementing a new screening tool: A qualitative study applying the Theoretical Domains Framework. J Clin Nurs. 2023;32(11–12):2867–79. 10.1111/jocn.16410. 35739640 10.1111/jocn.16410

[70] WMA. The World Medical Association. Declaration of Helsinki. JAMA. 2013. 10.1001/jama.2013.281053. 310(2091-94). 10.1001/jama.2013.281053 24141714

