# Supplementary material for: Frontline staff experiences of bridging dual diagnosis treatments – Determinants for implementing a cross-sectoral collaboration model
Source: Addict Sci Clin Pract. 2026 May 29;21:47. doi: 10.1186/s13722-026-00681-3 (PMC13221770; doi:10.1186/s13722-026-00681-3)
Supplement: Supplementary file 1 — Supplementary Material 1 [file 13722_2026_681_MOESM1_ESM.docx]

Appendix 1. A retrospective list of the most frequently used strategies for implementation of the SPOR model.

| **Used strategies according to the ERIC discrete implementation strategy compilation (Powell et al 2015)**  *(In alphabetical order)* | **Actions** | **Frequency /interval** |
| --- | --- | --- |
| Assess for readiness and identify barriers and facilitators | Pre-assessment with 14 interviews (Spring 2021)  Pre-Events (Summer 2021)  20 “difficult cases” collected and discussed with employees  Initiation of current CFIR study | Once  Three  3 months 2023  Data collection  Apr. - Oct. 2023 |
| Audit and provide feedback  + Facilitate relay of clinical data to providers | Dataset that monitors status and progress on selected variables presented at improvement meetings to clinicians and managers. | Monthly |
| Build a coalition | Involvement at several levels. Stakeholders were invited in early development and design phase.  Executive board established.  Formation of “Operations management group” (leaders+employees)  Resource persons (*see description under ‘Identify and prepare champions’) | N/A |
| Conduct educational meetings  + outreach visits | Outreach visits (info meetings and education) by project team at each site.  A manager or employee from each site visit collaboration sites. | Monthly  One visit at each site + if needed |
| Conduct local consensus discussions | Different meeting types endorsing an open discussion:  Coordination meeting with management in psychiatry  Improvement meeting with staff  Project team present at FACT- board meetings  Cross-sectoral improvement meetings for managers from both sectors  Coordination meetings with SUD treatment centers  Executive Board meeting  Dialogue meetings with employees | 2-4 weeks  4 weeks  Once a week  8 weeks  8 weeks  12 weeks  When needed |
| Develop and distribute educational materials | Pocket-size action cards, flyers and posters providing an overview of available contacts, treatment offers, procedures etc.  Distributed both physically at meetings and via e-mail. | ongoing |
| Facilitation | Project management team  present at least once a week in all teams  Close communication with all involved: On call and mail, daytime, mon-fri.  Conduct all meetings, write status notes, summaries, develop project material etc. | Daily |
| Identify and prepare champions  + Use train-the-trainer strategies | *Resource persons** (ordinary staff members with 5 days course in DD). Purpose: to increase the focus of DD among team members. | ongoing |
| Remind clinicians | Educational materials  Project team present  Managers and resource persons bring focus toward DD topics.  Screening incorporated in the electronic patient journal. | Ongoing  Weekly  Daily  Daily |
| Revise professional roles | “Cross-sector secretary” (medical secretary) in a coordinating role with access to both IT-systems to book network meetings. | Continuously |

Abbreviations: Consolidated framework for Implementation Research (CFIR), Substance Use Disorder (SUD), Dual Diagnosis (DD), Sammenhængende Psykiatri- og Rusmiddelbehandling (SPOR) (eng. Translation: Coherent Psychiatry and substance use treatment), Flexible Assertive Community Treatment (FACT), Denmark (DK).

* This function is not exclusive for SPOR, but part of standard FACT Implementation in the capital region of DK.
