# Supplementary material for: Frontline staff experiences of bridging dual diagnosis treatments – Determinants for implementing a cross-sectoral collaboration model
Source: Addict Sci Clin Pract. 2026 May 29;21:47. doi: 10.1186/s13722-026-00681-3 (PMC13221770; doi:10.1186/s13722-026-00681-3)
Supplement: Supplementary file 2 — Supplementary Material 2 [file 13722_2026_681_MOESM2_ESM.docx]

Table 2. Overview of interview participants (n=45)

| **Characteristics** | **Value** |
| --- | --- |
| Individual interviews  Group interviews, participants (n)  Nurses  Social workers  Addiction counselors  Psychologists  Medical Doctors  Occupational therapist  Physiotherapist  Nursing assistants  Other | 16  29  17  10  4  3  3  2  1  2  3 |
| Years in current workplace, mean (range) | 4.7 (0 - 16) |
| Years in profession, mean (range) | 13.8 (1 - 36) |
