# Supplementary material for: Frontline staff experiences of bridging dual diagnosis treatments – Determinants for implementing a cross-sectoral collaboration model
Source: Addict Sci Clin Pract. 2026 May 29;21:47. doi: 10.1186/s13722-026-00681-3 (PMC13221770; doi:10.1186/s13722-026-00681-3)
Supplement: Supplementary file 3 — Supplementary Material 3 [file 13722_2026_681_MOESM3_ESM.docx]

Appendix 3. A generic semi-structured interview guide.

Brief introduction about the study & purpose

Use of dictaphone

Anonymity, participation, and right to withdrawal

Intro questions:

Professional background? Current job title/function? Seniority at current workplace and in total?

**Regarding dual diagnosis in general:** *(Before SPOR)*

What are your previous experiences concerning patients with dual diagnosis?

What experiences have you had regarding cross sector collaborations to this patient group?

Approximately, how many patients with dual diagnosis would you say are in your team?

**General knowledge and attitude to SPOR:**

How long since you have been introduced to SPOR?

How long would you say that you have worked actively with SPOR in your team?

Is there a need for an intervention like SPOR? Why/why not?

Are there any advantages or disadvantages by being part of SPOR?

**The elements in SPOR:**

**Screening**

When do *you* think a patient has a substance use problem?

Do you use AUDIT/DUDIT on all patients? (other types?) If no, in which situations is it not performed?

What do you think of the AUDIT/DUDIT questionnaire?

How do you feel about asking a patient about his/her substance use?

How do you feel asking you patient about psychiatric illness? Do you use any screenings for that?

How often do you talk to your patients about the interaction between psychiatric illness and substance use?

**Motivation**

What are your experiences with motivating patients into treatment in the opposite sector? Please describe a successful and/or ‘failed’ case if possible.

Despite long term motivational work, have you tried that a patient refused treatment in the opposite sector? What did you do in that case?

Which tools do you use in the motivational work (MI, CBT, other methods)?

**Companionship**

Have you tried to physically follow any patients to the opposite sector? If yes, what was your experience? If no, what were the reasons for not doing so?

What does the companionship do for 1) the patient, and 2) you, professionally?

Are there any patients who benefit more from companionship than others?

**Coordination**

How does the cross-sector collaboration work with SPOR?

What is your experience with the cross-sector secretary?

Have you been part of any network meetings? If yes, what are your experience?

Is the degree of coordination satisfactory?

**Patients**

Do you experience that the SPOR-model meet patient needs? Is it suitable for *all* patients with dual diagnosis? Elaborate why/why not?

**Professional role and competencies**

Have you had any training/education in SPOR?

Do you need more education/training to be able to perform the elements in SPOR?

Do you perceive all elements in SPOR as part of your core responsibilities and professional tasks?

**Leadership**

Is SPOR prioritized and supported by management?

Do you have sufficient resources to implement SPOR?

**Change and future perspectives**

Do you believe that SPOR has changed your practices and working procedures?

With the implementation of SPOR, do you and your colleagues view differently at dual diagnosis? Do you address substance use/ psychiatric illness to a higher degree than before?

Has your collaboration with the opposite sector improved? Please elaborate why/why not.

Is there anything the project team should pay attention to in the future implementation work?
