# Supplementary material for: Frontline staff experiences of bridging dual diagnosis treatments – Determinants for implementing a cross-sectoral collaboration model
Source: Addict Sci Clin Pract. 2026 May 29;21:47. doi: 10.1186/s13722-026-00681-3 (PMC13221770; doi:10.1186/s13722-026-00681-3)
Supplement: Supplementary file 4 — Supplementary Material 4 [file 13722_2026_681_MOESM4_ESM.docx]

Appendix 4. Coding matrix with examples of the analytic process.

| Meaning unit | Condensed unit,  manifest content | CFIR domain | Latent interpretation |
| --- | --- | --- | --- |
| *It’s a huge strength with this collaboration, people experience the increased communication […] and coordination. I believe it makes a huge difference. Spg2* | Collaboration have strengthened communication and coordination | Innovation / relative advantage | With the implementation of SPOR people experience an improvement in a positive direction |
| *I have talked to some patients who did not want to discuss their substance use, because they thought that we would get angry with them, because it’s problematic in relation to their diagnostics. But when I ‘open’ the substance use talk and we talk about it, they become calm and actually said yes to substance use treatment.* Hba8 | Some patients did not want to talk about their substance use, thought staff would be angry. When they have the conversation after all, they feel calm. | Individual / innovation recipient | Prior experiences may form patients’ attitudes and reactions. Guards are up until they realize that it is allowed to talk about substance use in psychiatry. As recipients to the innovation, these experiences need to be addressed in the implementation process. |
| *I feel a significant difference between now and then… before we always had frustrations with patients with substance use - we were like “What are we gonna do with them? They should just quit! Just.. don’t do it!” Our focus wasn’t there, we had no dialogue about it, and there was no help. It was up to the individual employee […] how much attention it would get. And now, we keep them in trajectories longer and work towards reducing the use to an extent where it is possible to gain a clearer picture and be able to diagnose. hba1* | Patient’s substance use was not a focal point before. The general idea was that they should just quit. Random how much attention it received in opposition to now, where they are kept in treatment longer with the aim of reduction. | Inner context / culture | A cultural shift is developing in the psychiatric outpatient clinics, from abstinence as the primary goal to a harm reduction approach. |
| *Well, we have had a lot of frustrations with psychiatry, since we feel they ‘throw’ the patients back at us. They [psych] end treatment, nothing happens, and then we are on our own with the patient. What are we going to do with them? And they keep getting worse, we can only help them with their substance use. pr.blv2* | Frustrating that psychiatry terminates treatment and ‘throw’ patients back to substance use treatment when they consider people are still ill and in need of psychiatric treatment. | Outer context / partnerships & connections | Power relations are present. It is incomprehensible for staff from the municipality to grasp treatment standards and procedures in psychiatry, they feel left alone. To them, it is a person in need of help, who ends up being a ‘throwing ball’. Transparency in rejections could benefit collaborations. |
| *I think that implementation should be even more close to practice. What do I do when I experience this? Who can I call? What is the procedure? It can be made on a poster. Information material to hang in your office is always nice. Prblv 1* | Practice oriented information material would be nice to enhance implementation | Implementation process / assessing needs | Information materials such as posters, action cards or similar visuals act as reminders to support staff during implementation. Further, the need for specific examples and detailed how-to procedures reflects an insecurity calling for more training. |
