# Supplementary material for: Frontline staff experiences of bridging dual diagnosis treatments – Determinants for implementing a cross-sectoral collaboration model
Source: Addict Sci Clin Pract. 2026 May 29;21:47. doi: 10.1186/s13722-026-00681-3 (PMC13221770; doi:10.1186/s13722-026-00681-3)
Supplement: Supplementary file 5 — Supplementary Material 5 [file 13722_2026_681_MOESM5_ESM.docx]

Appendix 5. Barriers and facilitators expressed by outpatient psychiatry (P) and municipal substance use treatment (S) according to CFIR-domains.

|  | **Barrier** | **Facilitator** |
| --- | --- | --- |
| **The Innovation Domain** | Screening can affect relations (P,S) | Positive expectations of model potential (P,S) |
|  | Screening is too superficial (P,S) | Screening is quick and easy (P) |
|  | Model draws on known elements (P,S) | Model draws on known elements (P,S) |
|  | Assessing psychiatric illness is difficult (S) | Electronic screening available (P) |
|  | Motivation is time consuming (P,S) | The model is not too comprehensive (P,S) |
|  | Cultural practices take time to change (P,S) | Enhancing training in psychiatric assessment (S) |
|  | Negative staff attitudes on patient motivation (P,S) | Positive staff attitudes on patient motivation (P,S) |
|  | Lack of knowledge of the opposite sector (P,S) | Increased knowledge of the opposite sector (P,S) |
|  | Companionship is resource demanding (P,S) | ‘humanizing’ the opposite sector (P,S) |
|  | Clear role distribution is necessary with companionship (P,S) | Companionship increase safety for patients (P,S) |
|  | Companionship from psych🡪SUD happens more often than the other way (P,S) | Companionship has derivative positive effects for staff (P,S) |
|  | Network meetings are time- and resource demanding (P,S) | Network meetings increase the understanding for each other’s work (P,S) |
|  | Network meetings require preparation (P,S) |  |
|  | Network meetings can be overwhelming for the patient (P,S) |  |
|  | Difficult to coordinate when ending treatment (P,S) |  |
| **Individuals Domain**  **(recipients)** | Stigma in wording (P,S) | Empathy for patient group (P,S) |
|  | High degree of flexibility is needed (P,S) | Encounter with the system should not be an obstacle (P,S) |
|  | Patient motivation, resources and capabilities is fluctuating (P,S) |  |
|  | Target group complex (P,S) |  |
| **Inner Context Domain** | De-prioritizing due to busy practice (P,S) | Increased knowledge and competencies with SPOR (P,S) |
|  | some services are invisible to management (P) | Working towards a focus on reduction instead of abstinence (P) |
|  | Time and resources (P,S) | Slowly changing practices regarding rejection (P,S) |
|  | Avoiding topic of substance use to keep out of conflict (P) |  |
|  | Call for more knowledge on substance use treatment (P) |  |
|  | Call for more knowledge about psychiatric illness (psychosis) (S) |  |
|  | A top-down decision to implement (P) |  |
| **Outer Context Domain** | Double work (P,S) | Realizing double work is an eyeopener that unifies them (P,S) |
|  | Continuous communication problems (P,S) | Communication pathways improved (P,S) |
|  | Accessibility (P,S):  Opening and telephone hours in sud (P) High staff turnover in psy (S) |  |
|  | Ending treatment too soon (P,S) |  |
| **Implementation Process Domain** | Lack of knowledge despite implementation efforts (P,S) | educational efforts (P,S) -may increase |
|  | Amount of meetings (P,S) | Reminders (P,S) - may increase |
|  | Shift in involvement for employees (P,S) | Educational material (P,S) -may increase |
|  | Competing interventions implemented simultaneously (P,S) | The project team (P,S) |
|  |  | The opportunity to meet and discuss in forums (P,S) |
|  |  | Management support (P,S) |
